# Identifying Migration Routes of Wild Asian Elephants in China Based on Ecological Networks Constructed by Circuit Theory Model

**DOI:** 10.3390/ani13162618

**Published:** 2023-08-14

**Authors:** Xin Jiang, Hong-Jie Liu, Zhi-Yun Jiang, Ru-Ping Ni

**Affiliations:** School of Geography, South China Normal University, Guangzhou 510631, China; xinjiang@m.scnu.edu.cn (X.J.); 2022022795@m.scnu.edu.cn (R.-P.N.)

**Keywords:** circuit theory model, ecological corridor, ecological pinch point, conservation, Asian elephant (*Elephas maximus*)

## Abstract

**Simple Summary:**

In March 2020, a group of wild Asian elephants suddenly roamed north from China’s Xishuangbanna National Nature Reserve and subsequently arrived in Kunming City, Yunnan Province, which has aroused widespread concern in the society. Based on this event, this study attempted to identify the possible migration routes of Asian elephants according to the existing land conditions and the movement habits of Asian elephants if wild Asian elephants in China migrated again. It was found that the areas with a high probability of migration by Asian elephants were mainly concentrated in the gently undulating shrubs, bamboo forests, and grasslands. Moreover, dense forests with steep slopes and high altitudes, cultivated land, and construction land are less likely to be passed by Asian elephants. Our findings can not only provide valuable insights into suggestions and solutions for the current conservation of wild Asian elephant species but also be beneficial in biological protection and biological reserve planning.

**Abstract:**

Humans overlap with Asian elephants, resulting in frequent costly human–elephant conflicts, which disturb and even threaten local residents. In this study, we treat provincial and national nature reserves where Asian elephants still exist and other alternative habitats suitable for Asian elephants in southern Yunnan, China, as ecological patches. By using this approach, we can treat the terrain and surface state factors that hinder the migration of Asian elephants as a form of ecological resistance surface. We can then use a circuit theory model and remote sensing data to construct an ecological network, which allows us to identify ecological corridors and ecological pinch points. Herein, the possible migration routes of wild Asian elephants were identified. The main results are as follows: (1) In the study area, dense forests with steep slopes and high altitudes, cultivated land, and building land have greater migration resistance, while the gently undulating shrubs, bamboo forests, and grasslands far away from the city have less migration resistance. (2) There are three ecological corridor groups in the study area, mainly composed of shrub and grassland. The ecological corridors identified in this paper are the most likely migration routes of wild Asian elephants in China, and areas with higher simulated current densities reflect a higher probability of Asian elephants passing through. (3) According to the analysis, the ecological pinch points in the study area are 602 km^2^ in total, and woodland and grassland account for 89.2% of the total ecological pinch area. The areas where the pinch points are located have a high probability of Asian elephants passing through and a narrow space. Our findings can provide suggestions and solutions for the current conservation of wild Asian elephant species, alleviate human–elephant conflicts, promote the harmonious coexistence between humans and nature, and provide reference for biological protection and biological reserve planning.

## 1. Introduction

The Asian elephant (*Elephas maximus*) is a first-class protected animal in China, which can be found in most areas south of the Yellow River Basin, and is listed as an endangered species by the International Union for Conservation of Nature (IUCN). The species is threatened by climate change and various human activities (e.g., deforestation), and consequently, the distribution of Asian elephants in China has retreated southward at a rate of 0.5° latitude per century [1]. Asian elephants are migratory mega herbivores requiring a large space to live—space that has diminished considerably in recent years [2]. The acceleration of urbanization and industrialization has rapidly eaten away at the available habitat, leaving the Asian elephant on the brink of extinction [3,4]. At present, there are only about 300 wild Asian elephants left in China, mainly distributed in the southern part of Yunnan [5]. Habitat loss and fragmentation for Asian elephants are mainly due to massive deforestation [6]. Human production and living spaces have replaced forests in many areas [7]. Traditional migration routes of Asian elephants are now cut off, and human settlements and Asian elephant ranges have begun to gradually overlap over the last two centuries [8]. Understandably, human–elephant conflicts occur frequently, and there were 27 deaths and many injuries from 2011 to 2019 [9]. Human–elephant contact is not only expensive but also interferes with and threatens the daily activities and safety of local residents. Accidents related to Asian elephants have become a serious local problem. Therefore, effective measures to alleviate human–elephant conflicts should strengthen the quality restoration of Asian elephant habitats and foraging areas on the basis of protecting the rights and interests of rural settlements and urban built-up areas. However, the restoration of Asian elephant habitat quality is usually measured on a 10-year scale [10]. Designing available ecological corridors for Asian elephants is critical to guide Asian elephants to suitable areas in case of reoccurrence of migration. Predicting the movement route of the elephant herd is the basis for promoting the protection of Asian elephants and alleviating human–elephant conflicts. However, current studies have focused on the assessment of the habitat quality of Asian elephants and the factors affecting habitat quality or the society’s attitude and awareness of the human–elephant conflict [1,11]. The limited understanding of the prediction of migration routes has hindered the development of mitigating human–elephant conflicts and protecting wild Asian elephants.

Circuit theory, derived from physics, was applied by Mcrae and Beier to study the gene flow in heterogeneous landscapes, arguing that circuit theory can be used to predict the migration path of biological populations and the probability of successful dispersal [12]. The charge in the circuit has the characteristics of a random walk, and the spillover of species is also highly random. The circuit theory model connects circuit theory and motion ecology through random walk theory. At present, the circuit theory is often used in the research on ecological sustainable development and long-term construction and the study of ecological networks based on biodiversity [13,14]. Little research has been conducted leveraging this approach to identify species migration and movement ecology with an eye toward conservation in a human–wildlife context. The “ecological patch-ecological resistance surface-ecological corridor” research paradigm is often aimed at providing effective and systematic means of solving ecological problems in land space planning and the ecological restoration of land space and is committed to the application of the ecological network and ecological security pattern [15,16]. The general ecological significance of this paradigm is reflected in the research, and ecological network construction for specific species is considered slightly narrow and insufficient to date.

In March 2020, a group of wild Asian elephants roamed north from China’s Xishuangbanna National Nature Reserve and arrived in southern Kunming in early June 2021. The event has attracted widespread international attention, largely because no such long-distance migration of Asian elephants has occurred in the past half-century [2]. Elephant migration is a complex ecological process that varies in distance, timing, duration, and drivers. At present, there are many controversies about the reasons for elephant migration in academia. This study focuses on the discussion on how to scientifically identify the migration routes of Asian elephants. Based on the existing situation and the premise of promoting the protection of Asian elephants and mitigating human–elephant conflicts, this paper uses a circuit theory approach and adopts the research paradigm of “ecological patch-ecological resistance surface-ecological corridor” to identify the migration routes of Asian elephants.

## 2. Materials and Methods

### 2.1. Study Area and Data Sources

**Figure 1:**
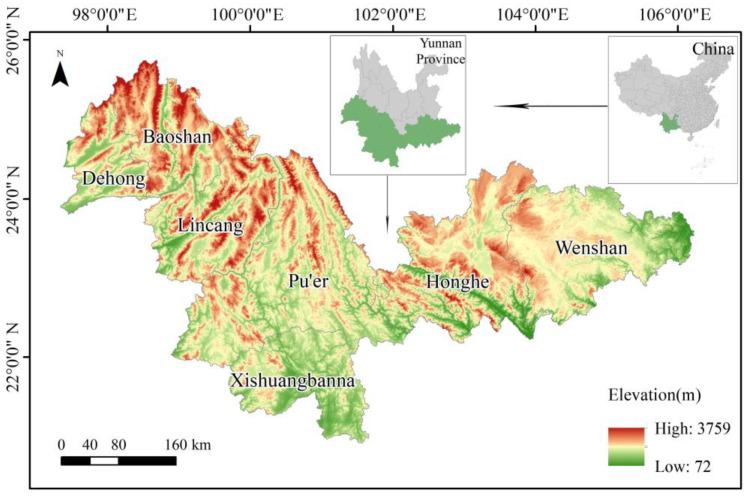
Location and topography of the study area.

The study area is the cities and autonomous prefectures where the main protection objects include Asian elephants or habitats suitable for the survival of Asian elephants in the provincial and national nature reserves in the “National List of Nature Reserves (2015)”. In our survey of these sites, we included Baoshan, Lincang, Pu’er, Dehong, Xishuangbanna, Honghe, and Wenshan, a total of 3 cities and 4 autonomous prefectures (Figure 1). These locations are located within the south of Yunnan Province, China, between 21°05′~25°50′ north latitudes and 97°29′~106°14′ east longitudes, bordering Myanmar, Laos, and Vietnam, with a total area of 181,128 km^2^ and an elevation of 72~3759 m. The study area is located in the low-latitude monsoon climate zone, which is the area most affected by the southwest monsoon in China. The study area is rich in habitat types and is considered a global biodiversity hotspot [17].

The data used in this study consisted mainly of land use, terrain, administrative boundary nature reserve, and Asian elephant historical early warning points. Among these data sources, land-use remote sensing monitoring data (30 m × 30 m), digital elevation model (DEM) data (90 m × 90 m), and administrative boundary vector data of the study area were obtained from the Data Center for Resources and Environmental Sciences, Chinese Academy of Sciences (https://www.resdc.cn/, accessed on 6 March 2023). The data for nature reserves were obtained from the website of the Central People’s Government of the People’s Republic of China (https://www.gov.cn/, accessed on 6 March 2023). The historical early warning points of Asian elephants were obtained from the Asian Elephant Early Warning System (http://elephant.noahsark.org.cn/#/, accessed on 1 August 2023).

### 2.2. Methodology

**Figure 2:**
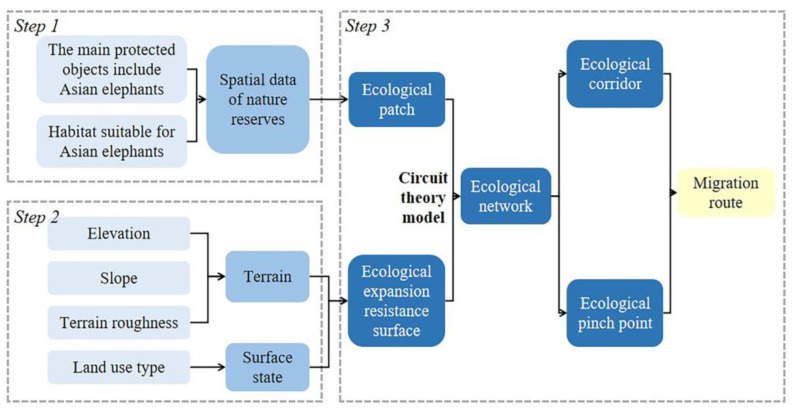
Flowchart of the analysis research procedures in this study.

As described, we focused our research on identifying potential areas where we could connect the “ecological patch-ecological resistance surface-ecological corridor” paradigm. In this context, areas with Asian elephants are considered type I ecological patches, and protected zones with suitable habitats for Asian elephants are taken as type II ecological patches. By synthesizing the terrain and surface state factors that hinder the ecological flow of Asian elephants we could establish an ecological resistance index system. By then applying circuit theory, we can identify an ecological network, set thresholds to extract ecological corridors and ecological pinch points, and produce a model of potential migration pathways (Figure 2).

#### 2.2.1. Identification of Ecological Patches (Step 1)

The facilitation of ecological functions and the preservation of ecological integrity can be enhanced by the presence of high-quality ecological patches [17]. For this purpose, ecological patches were designated as provincial and national nature reserves, as these areas predominantly harbor wild Asian elephants in southern China [5]. Based on previous studies [1,18], this study classifies nature reserves that primarily focus on Asian elephants as ecological patch I, while nature reserves with high-quality Asian elephant habitat evaluation are classified as ecological patch II. This study focuses on wild Asian elephants in China. Initially, migration begins from ecological patch I, and as biological flow and the spread of Asian elephants occur, both ecological patch I and ecological patch II become equally significant. The approach examined in this study aligns with the prevailing circumstances of China’s swift urbanization and the underlying purpose of establishing nature reserves.

#### 2.2.2. Construction of Ecological Resistance Surface (Step 2)

The accessibility and connectivity between ecological patches can be judged by setting the minimum expansion resistance value, since species need to overcome certain resistance for spatial movement [19]. The setting of the resistance value of ecological expansion should consider various factors influencing the dispersal behavior of specific wild species [20]. However, there is currently no accepted standard for setting resistance values [21]. It is simplest to envision the “resistance” value as a holistic estimate of how difficult it is for a given species to move through a given corridor. Much as the electrical resistance of an object is a measure of its resistance to the flow of current, these resistance values are estimates of the difficulty associated with navigating an ecological corridor [22,23]. The construction of a generalized ecological corridor then might be considered a more comprehensive assessment that takes into account the average difficulty any species may have traversing the corridor [14,17]. This study comprehensively analyzes the preferred distribution landscape types of wild Asian elephants in China and the geographical characteristics of spillover and constructs comprehensive resistance factors by synthesizing terrain surface state factors. Resistance values are often estimated using field surveys of topography and terrain, as well as animal tracking data. Estimation of resistance values might also consider niche factor analysis, though we do not attempt that here [24,25,26,27] (Table 1). Herein, “ecological resistance” is a product of four factors: elevation, slope, terrain roughness, and land-use type. Resistance values reflect the ease with which Asian elephants traverse different terrains and landscapes. The lower the resistance value, the more likely Asian elephants are to diffuse into the environment and the higher the accessibility and circulation of Asian elephant ecological flow. The higher the resistance value, the stronger the resistance to Asian elephant spillover migration. The terrain and landscape constitute rigid constraints on Asian elephant spillover; the potential range of the resistance value is regarded as infinite, and it is generally set to be large enough in the actual assignment process. To optimize computing efficiency, resistance values are capped at 1000 following [25,26,27].

Asian elephants are huge in size, with a female shoulder height of 2.24–2.54 m and a weight of 2720–4160 kg and a larger male shoulder height of 3.2 m and a weight of 5400 kg [28]. The size and physiological structure determine that it is difficult for Asian elephants to traverse steep and undulating areas. Consequently, this study considers both slope and terrain roughness resistance factors. Altitude also has a certain impact on the spillover migration of Asian elephants, but within the study area, altitude is not the dominant factor. Instead, migration may be impacted by habitat suitability. Wild Asian elephants in China prefer to live in bamboo forests, bamboo-broadleaved mixed forests, grasslands, or shrubs. They have strong adaptability to habitats with rich understory vegetation but weak adaptability to cultivated land and impermeable surfaces affected by human activities. Asian elephants often travel near water sources for food, swimming, and bathing. Asian elephants usually choose to cross shoals or small streams. In order to avoid being washed away or submerged by the current, Asian elephants generally do not choose to cross large rivers with rapid currents.

#### 2.2.3. Identification of Ecological Network (Step 3)

(1) Ecological network identification

Circuit theory connects circuit theory and motion ecology through random walk theory [29]. The model treats the landscape as a conductive surface and simulates the movement process of species as similar to the random flow of electrons. In this fashion, we can calculate the magnitude of the current between each ecological patch and consider all possible paths in the landscape [30]. The formula for calculating the current density between two nodes is

(1) $$I=\frac{V}{R_{eff}}$$

In the formula, *I* is the current through the conductor, *V* is the voltage measured through the conductor, and *R_eff_* is the effective resistance. The effective resistance *R_eff_* can reflect the spatial separation between nodes. The voltage *V* represents the probability that a random walker leaves any node and successfully reaches a given target node (i.e., the probability of successful diffusion down a corridor). The branch current value *I* can reflect the ecological flow through the resistance section of the branch, which can be used to predict the movement probability of the ecological flow on the corresponding node or path.

(2) Ecological corridor extraction

Ecological corridors are channels that ensure energy and material flow between important ecological patches. When the corridor has a certain width and habitat quality, it will ensure the realization of ecological processes and ecological functions [31]. Put simply, a corridor is an area through which organisms may reliably pass. In some areas, the current density is extremely low, and the possibility of Asian elephants passing through is extremely small. Conversely, if a connection is identified where current density in the ecological corridor area is higher, the connectivity between the patches is higher [22,23]. This study employs the natural break (Jenks) classification method to ensure the ecological corridor’s significant connectivity and high current density [32]. The current density is divided into ten categories, with the first two categories (0.003) serving as the threshold for extracting the ecological corridors. These corridors represent the identified migration routes of wild Asian elephants in China. The zonal statistics tool in the ArcMap10.6 toolbox is utilized to conduct extraction queries and obtain area statistics for each land-use type.

(3) Ecological pinch point identification

Circuit theory also introduces a concept known as an “ecological pinch point”. When an area in the model has a high current density in the ecological network, the possibility of biological movement is high, and it has strong irreplaceability [33,34]. Combining the minimum resistance model and the circuit theory model was used to identify ecological pinch points [35]. However, when there is large resistance value in the surrounding area, biological movement is compressed in a relatively narrow range, resulting in a “pinch point” through which a large amount of traffic is funneled through a relatively small aperture in the model network [36]. In this paper, a 10 km buffer zone is set for ecological patch I. The natural breakpoint method is used to divide the current density into three levels, and the area in the buffer zone with the highest current density is considered an ecological pinch point.

## 3. Results

### 3.1. Ecological Patches

**Figure 3:**
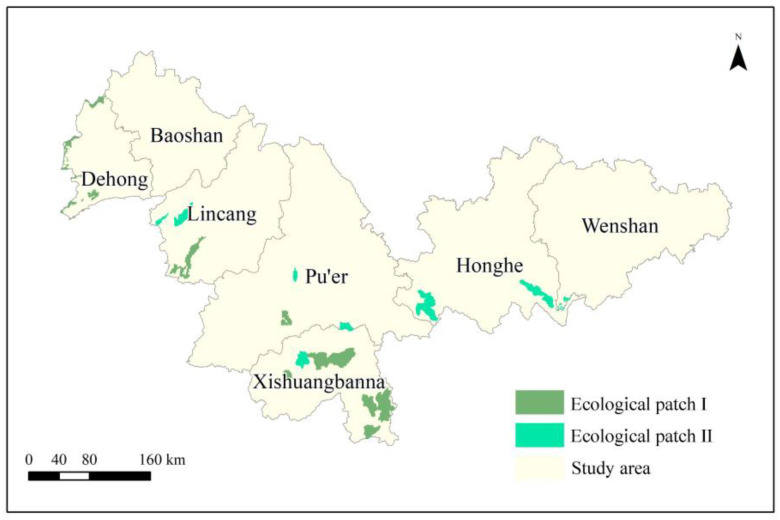
Distribution of ecological patches in the study area. Ecological patch I pertains to the nature reserves primarily safeguarding Asian elephants as the main protected species, while ecological patch II designates the nature reserves exhibiting a high evaluation of Asian elephant habitats.

There are 28 ecological patches totaling 5612.18 km^2^, accounting for 3.1% of the total surface of the study area. Most sources are distributed in the west of Dehong, the southwest of Lincang, central and southern Pu’er, Xishuangbanna, southwestern and southeastern Honghe, and southwestern Wenshan (Figure 3).

### 3.2. Ecological Resistance Surface

**Figure 4:**
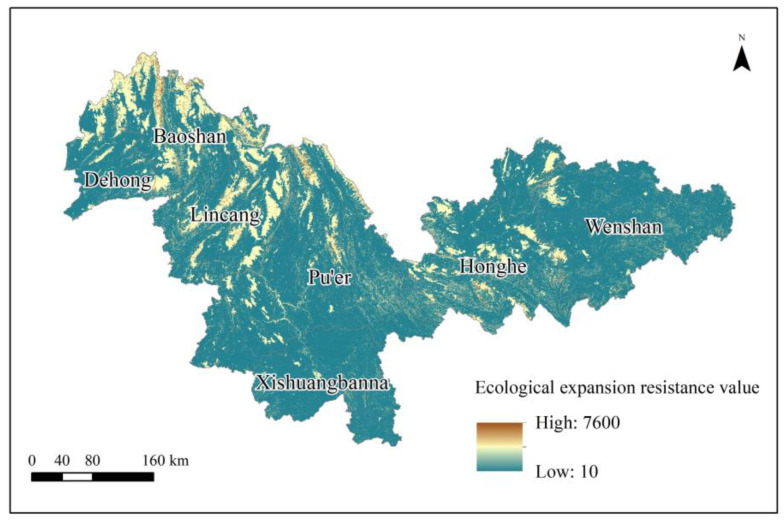
Resistance surface of ecological expansion in the study area.

The equal-weight superposition of four factors that hinder the spillover of wild Asian elephants in China is used to obtain the resistance surface of ecological expansion in the study area (Figure 4). Overall, the average ecological expansion resistance value in the study area is 545, the highest value recorded is 7600, and the lowest value recorded is 10. Extremely resistant surfaces were mainly concentrated in dense forests, cultivated land, and construction land with high altitudes and large slopes, such as Dehong, Baoshan, Lincang, the north of Pu’er, Honghe, and the northwest of Wenshan. The absolute elevation and relative elevation of this type of area are large, human activities are relatively intensive, and the restrictions on the spillover of Asian elephants are relatively high. In contrast, shrubs, bamboo forests, and grasslands far away from cities with low absolute and relative elevations are considered a superior ecological environment less affected by human activities, and the resistance to ecological expansion is small.

### 3.3. Ecological Network

**Figure 5:**
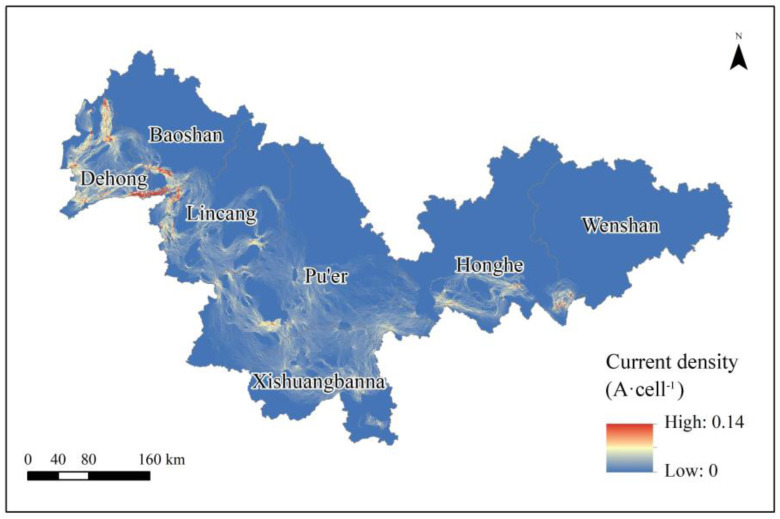
Distribution of current density in the study area.

The ecological network is identified through the circuit theory model, and currents of different densities connect each ecological patch in series to form a network appearance (Figure 5). Unsurprisingly, ecological corridors are identified and found to be mainly composed of shrub and grassland. The areas with higher current density in the ecological network are mainly distributed in the west and south of the study area, while the north and east have lower current density.

### 3.4. Ecological Corridor

**Figure 6:**
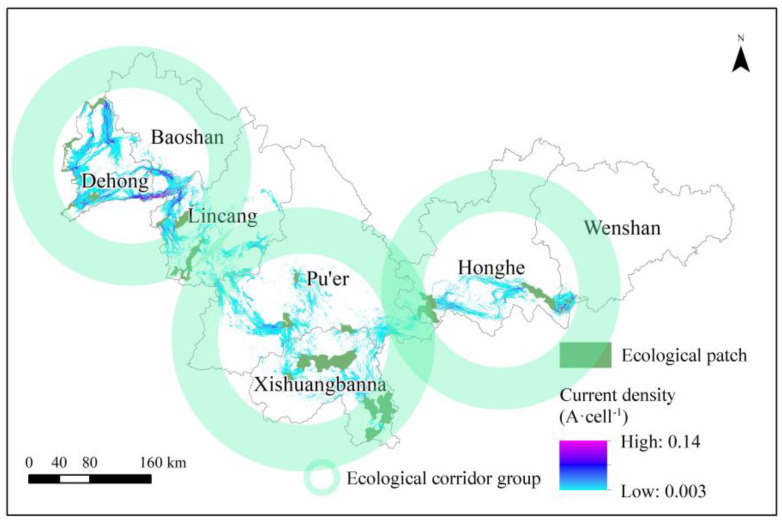
Distribution of ecological corridors in the study area.

Based on the random walk characteristics of the circuit theory model, we identified multiple possible migration pathways of wild Asian elephants in China. These routes could be largely divided into three ecological corridor groups (Figure 6). Areas with higher current densities reflect higher probabilities of Asian elephants passing through, which belong to areas with higher passing levels. The distribution of ecological corridors in the south and west is relatively dense. Forests and grasslands collectively constituted 84.3% of the overall extent of ecological corridors, predominantly shrubland and high-coverage grassland. These findings indicate that the ecological conditions along the migration routes of elephant herds are advantageous, thereby facilitating their migratory behavior. This conclusion is drawn from the observations that the environmental factors, including vegetation cover and water resources, align with the biological requirements of elephants.

### 3.5. Ecological Pinch Point

**Figure 7:**
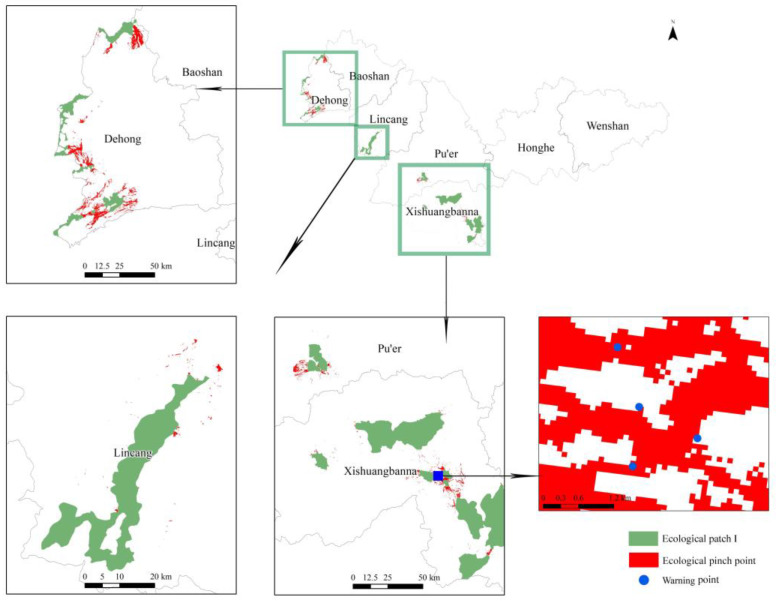
Location of ecological pinch points and warming points in the study area. The warning points were obtained from the Asian Elephant Early WarningSystem (http://elephant.noahsark.org.cn/#/, accessed on 1 August 2023).

We identified a series of ecological pinch points in Dehong and Xishuangbanna, the southwest of Lincang, and southern Pu’er, with a total area of 602 km^2^ (Figure 7). Forest and grassland cover account for 89.2% of the total area within ecological pinch points, predominantly shrubland and high-coverage grassland. These ecological pinch points have a higher probability of being traversed by Asian elephants. Therefore, the villages and residential areas around the ecological pinch point need to set up defensive measures, and most ecological pinch points can strengthen ecological conservation work. The historical early warning records of Asian elephants were utilized to validate the efficacy of ecological pinch points in identifying their locations. The result (Figure 7) displayed that certain warning points of Asian elephants align well with the ecological pinch points identified in this study. It should be pointed out that the Asian elephant early warning system is exclusively deployed in Xishuangbanna, the region with the highest concentration of Asian elephants, and the monitoring equipment is primarily situated within the nature reserves.

## 4. Discussion

### 4.1. Validity of Circuit Theory Model in Identifying Migration Routes of Asian Elephants

Movement models that incorporate random walks have been shown to be highly consistent with real-world patterns of animal dispersal [37,38]. Consequently, the migration routes identified herein may represent real suggestions and solutions for the current conservation of wild Asian elephant species. The improved ability to predict the movement of this endangered species will alleviate human–elephant conflicts, promote the harmonious coexistence between humans and nature, and provide reference for biological protection and biological reserve planning. Lin et al. [39] collected the existing distribution data of wild Asian elephants in Xishuangbanna and the distribution data of towns, villages, and roads in the wild and the habitat types and migration habits preferred by wild Asian elephants, using GIS technology to explore how to choose the appropriate corridor belt route. Unfortunately, our conservation efforts are limited, and due to the neglect of the high randomness of biological activities, this research result can only provide reference for the construction of an ecological corridor in the Xishuangbanna National Nature Reserve. Similarly, Buchholtz et al. [40] used African Wildlife Tracking (AWT) collars to obtain GPS data on elephant herds. This method has certain requirements for the number of elephant herd samples, and different biological protection policies in different regions make it difficult to popularize the case study method. Both of these efforts are limited by the challenges of monitoring animal tracking and consequently have limited impact.

Often, resistance values are estimated through costly field surveys or expensive and long-term animal monitoring projects [41,42,43]. Our model design allows us to simulate random animal movement practically and easily without these costly challenges. The circuit theory model used in this paper can cheaply and practically be used to alleviate human–elephant conflicts with only a small amount of publicly available data. Furthermore, we can ground truth the efficacy of our model by testing if our surface-resistance approach would identify existing conservation tools; for example, the aforementioned Xishuangbanna National Nature Reserve planned by Lin et al. [39] is identified in our model as a high-quality corridor. Unsurprisingly, the corridor is included in the higher current density area of the ecological network identified in this study. The data involved in this study are easily accessible and rely on a well-established circuit theory. Moreover, we found that several of the “ecological pinch points” identified in this study corresponded to locations reporting human–elephant conflict [44]. We hope to show that these approaches are suitable for extension to the prediction of the migration routes of wild Asian elephants and other species in other regions.

Previous studies have integrated landscape connectivity [44,45], morphological spatial pattern analysis [46], and climate refugia [47] into the construction of Asian elephant ecological corridors, which are resource-intensive and more suitable for providing reference for long-term planning. Li et al. [48] assessed the risk of human–elephant conflicts based on an ecological-niche factor analysis model, with the risk zone accounting for 22.77% of the total area of Xishuangbanna. However, implementing the recommendations to reduce risk in these areas is challenging. By comparison, the method proposed in this paper has high feasibility and practicability in implementation on a small time scale and therefore holds greater practical significance.

The conservation of these critically endangered animals is paradigmatic of our global struggle with human–wildlife boundaries. While the application of circuit theory to animal movement analyses does require a somewhat subjective estimate of “resistance”, it is also broadly applicable and can produce reliable results in a number of systems, as we show in this study on Asian elephants. The best way to improve the rigor of these general models is to continue to collect in-field data and foster collaboration between field scientists and modelers. Future efforts should strive to do so.

### 4.2. Recommendations for Future Conservation of Asian Elephant Population

Rapid economic and population growth has put enormous pressure on wild animals and their habitats. When human production and living areas overlap with wild animal habitats, conflicts between humans and wild animals are inevitable [49]. Multiple studies have shown that the frequency of conflicts and economic losses between humans and wildlife is increasing [40,50]. Dealing with the relationship between humans and wildlife and establishing mechanisms for coexistence between them is a challenging task [51,52]. In the study area, human–elephant conflicts occur frequently, and accidents related to Asian elephants have become a serious local social problem [9]. This paper suggests building electric fences, anti-elephant ditches, and other facilities in residential areas near the ecological pinch to reduce the risk of human–elephant conflicts. We can use the data in this study to identify where food sources for Asian elephants may be placed in the research area or to identify where to lay infrared trigger camera alarm systems and set up iron fences, beehive fences, and Aversive Geofencing Devices around the villages within the range of the ecological network [53,54]. In order to fundamentally reduce the intensity and frequency of human–elephant conflicts and promote the coexistence of humans and Asian elephants, researchers and relevant departments need to strengthen the research on the ecology of Asian elephants and the relationship between human social development and the survival of Asian elephants. Studies of the migration of Asian elephants in terms of habitat quality, population dynamics, natural food sources, ecosystem integrity, monitoring the quality of ecological corridors by remote sensing techniques, and integrating dynamic analysis of the habitat are urgently needed [55,56]. Prioritizing the restoration of degraded habitat and feeding areas for Asian elephants is critical to addressing human–elephant conflict. By restoring these habitats and providing suitable feeding grounds, we can encourage Asian elephants to remain in protected areas, thereby reducing instances of conflict with humans. However, the improvement of habitat quality requires a certain time cost. If Asian elephant migration occurs again in the short term, we can also guide Asian elephants to natural reserves suitable for survival. In this context, this method has important research significance and practical application value in theory.

## 5. Conclusions

Herein, we used a circuit theory model to extract ecological corridors and ecological pinch points in the migration and dispersal of the critically endangered wild Asian elephant. Our results found that the average ecological expansion resistance value in the study area is 545, the highest value is 7600, and the lowest value is 10. Dense forests, cultivated land, and developing land with higher altitudes and steep slopes have greater resistance to ecological expansion, while shrubs, bamboo forests, and grasslands that are far away from cities and have low absolute and relative elevations have small resistance to ecological expansion. We identified three ecological corridor groups in the study area, mainly composed of shrub and grassland, which conforms to the actual behavioral patterns of Asian elephants and exhibits a mesh-like distributional pattern. The ecological corridors identified in this study represent possible migration routes for wild Asian elephants in China. The areas with higher current densities reflect a higher probability of Asian elephants passing through. We also identified key ecological pinch points representing 602 km^2^ of the total study area; woodland and grassland account for 89.2% of the total ecological pinch area. Pinch points represent narrow areas with a high probability of Asian elephants passing through. Residential areas near the ecological pinch point can be equipped with defensive measures to avoid human–elephant conflicts, and woodland and grassland can be protected to enhance the guidance of the migration route of Asian elephants. The migration routes and ecological pinch points of wild Asian elephants identified in this paper can provide methods and suggestions for the current conservation of the wild Asian elephant, alleviate human–elephant conflicts, promote the harmonious coexistence between humans and nature, and provide a basis for short-term biological protection.

## Figures and Tables

**Table 1:** Resistance factor and its assignment.

Resistance Factor Type	Factor of Resistance	Class of Classification	Value of Resistance
Terrain	Elevation (m)	<1000	10
		1000–1500	50
		1500–2000	100
		>2000	10,000
	Slope	<10	10
		10–15	50
		15–30	100
		>30	10,000
	Terrain roughness	<1.1	10
		1.1–1.2	50
		1.2–1.3	100
		>1.3	10,000
Surface state	Land-use type	Shrubbery, grassland, sparse woodland	10
		Forest, bare land, dry land	100
		Swamp, river beach, rural settlement	200
		Other woodland, paddy field, bare rock land	400
		Urban land, other construction land, river canal, lake, reservoir, permanent glaciers, snow	10,000

Note: Land-use type as defined by the Chinese National Land Use/Cover Classification System (CNLUCC) based on multi-period remote sensing data.

## Data Availability

Data will be made available on request.
